# Comparing Pharmacological and Nonpharmacological Interventions for Alleviating Preoperative Anxiety in Pediatric Surgical Patients: A Randomized Controlled Trial in Pakistan

**DOI:** 10.7759/cureus.82502

**Published:** 2025-04-18

**Authors:** M Asghar Ali, Muhammad Hassan Khan, Bushra Salim

**Affiliations:** 1 Department of Anesthesiology, Aga Khan University Hospital, Karachi, PAK; 2 Department of Anesthesiology, Letterkenny University Hospital, Letterkenny, IRL

**Keywords:** non-pharmacological interventions, operative, pediatrics, pharmacological interventions, preoperative anxiety, surgical procedures

## Abstract

Introduction

Preoperative anxiety can prolong the induction of anesthesia and postoperative recovery, increase the risk of postoperative delirium, increase pain, and increase analgesic use. Pharmacological interventions are associated with increased cost to the hospital, potential surgical delay while waiting for the medication to take effect, and delayed discharge from the recovery room, while nonpharmacological modalities, including electronic gadgets, are cost-effective, noninvasive, and carry a low risk for adverse effects. This study aimed to compare pharmacological and nonpharmacological interventions (use of technology) for alleviating preoperative anxiety in children undergoing general anesthesia in Pakistan. We hypothesize that digital distraction will reduce preoperative anxiety more effectively than oral midazolam.

Methods

A randomized controlled trial was conducted on 106 children scheduled for elective surgery. Written informed consent was obtained from the patient/next of kin. Patients were assigned to one of two groups by a computerized list. The control group received oral midazolam 0.5 mg/kg at least 30 minutes before surgery, and the interventional group was distracted by using digital devices (tablets). Children's perioperative anxiety was assessed using the Modified Yale Preoperative Assessment Scale in the preoperative holding area and the OR just before induction, with higher scores showing more anxiety. The Shapiro-Wilk test was applied to examine the normality of average scores at preop and inside the OR. The Mann-Whitney U test was used to compare the control and interventional groups.

Results

The analysis included a total of 106 pediatric patients. The primary endpoint, measured by anxiety levels using the Yale Preoperative Anxiety Scale, was compared between the two groups in the preoperative holding area and just before induction. In the holding area, the median scores were 46.67 (IQR 26.6) for Group A and 28.33 (IQR 23.33) for Group B. Just before induction, the median scores were 46.67 (IQR 27.50) for Group A and 23.33 (IQR 10.0) for Group B. The maximum preoperative scores were 70.0 (median 46.67, IQR 26.67) in Group A and 78.33 (median 28.33, IQR 23.33) in Group B. At induction, Group A had a maximum score of 68.33 (median 46.67, IQR 27.50), while Group B had a maximum score of 55.0 (median 23.33, IQR 10.0).

Conclusion

The results indicate that distraction techniques can be considered an alternative to traditional pharmacological premedication for children undergoing elective surgery.

## Introduction

The process of preparing for surgery and undergoing general anesthesia can be extremely stressful for children because of their dread of pain, their unfamiliar surroundings, and the presence of medical professionals [1]. In certain situations, the child may run away from the anesthesia and the surgical staff [2]. Reactions of dread or rage, feelings of powerlessness, increased motor tone, and aggressive behavior are some examples of this anxiety [2]. Preoperative anxiety can worsen pain, lengthen the time it takes to induce anesthesia, slow down the healing process after surgery, raise the risk of postoperative delirium, and increase the need for analgesics [2,3]. Preoperative anxiety can negatively affect children's daily functioning and is linked to several maladaptive behaviors (such as eating disorders, sleep difficulties, separation anxiety, nightmares, and behavioral conflicts with parents) [4]. It can have a negative impact on the child's response to future medical care [4]. Since about 60% of children experience anxiety before surgery, and because of the problems that cause anxiety, researchers are looking for ways to treat or prevent preoperative anxiety in children and possibly reduce its negative effects after surgery. Such interventions include nonpharmacological and premedication interventions with sedation drugs before surgery [3,4].

Pharmacological interventions have certain drawbacks, as they cause sedation and need proper monitoring; there can be delays in administration, causing a delay in surgery and hence a prolonged stay in the recovery room and an increased length of hospital stay [4,5]. Nonpharmacological interventions, for example, the presence of a parent at induction, educational and behavioral strategies, and distraction of the children using technology, either smartphones or other digital devices, can be used to alleviate the child's anxiety without any increase in the potential cost and no adverse effects. These methods are cost-effective, minimally invasive, and carry a low risk of adverse effects, contributing to their continued and widespread implementation [6,7].

This study aimed to determine the gaps in the literature regarding the effects on pediatric preoperative anxiety of pharmacological and nonpharmacological therapies, such as the video distraction method. Compared to the pharmacological group, we hypothesized that the nonpharmacological intervention of video distraction would reduce preoperative anxiety in pediatric patients undergoing general anesthesia.

The main objective of the study was to compare pharmacological and nonpharmacological interventions (use of the digital device for video distraction) for alleviating preoperative anxiety using the Modified Yale Preoperative Anxiety Scale (mYPAS).

## Materials and methods

This prospective single-blind (outcome assessor) randomized controlled study was conducted in the operating room (OR) of a tertiary care hospital. The study obtained approval from the Ethical Review Committee of Aga Khan University (approval no: 2022-7684-22625) and Clinical Trials Unit (CTU, ID: NCT05469412, registered at ClinicalTrials.gov), in compliance with Good Clinical Practice (GCP). Patient confidentialities were maintained.

According to Cumino et al., children's anxiety at the holding area time point was 23.8% in the control group and 4.8% in the suggested intervention group after reduction [16]. With 80% power of the test, a 5% level of significance, and a confidence interval of 95%, the required sample size was 53 (children) in each group. Written informed consent was obtained from the parents or guardians of the children in the preoperative clinic. Assent was also taken from the older children. All children who underwent elective surgery, both as inpatients and as day cases, and fell under the age group of two to 18 years were included. The patients who had previous exposure to surgery, developmental delay, significant visual/hearing problems, chronic illness or pain, any physical or mental disability, or had analgesic /sedative infusions were excluded from this study. Patients were assigned to one of two groups (A and B) using computer-generated random assignment using opaque envelopes for allocation concealment.

Control group

Standard management combined with pharmacological intervention (oral midazolam 0.5 mg/kg) administered at least 30 minutes before surgery (maximum 20 mg).

Interventional group

Standard management was combined with distraction using technology (cell phone or tablet). The digital device was given to the child in the preoperative area until he/she moved to the OR, and they were asked to choose whatever cartoon, poem, or nursery rhyme they wished to watch.

One of the parents accompanied the child to the OR in both groups until induction. A trained data collector who was not a part of the study assessed the anxiety level by using the mYPAS in the preoperative holding area and the OR just before induction. This mYPAS, an observational instrument, quantifies children's anxiety. The instrument contains 27 items in five categories that indicate preoperative anxiety in children: activity, emotional expressivity, state of arousal, vocalization, and use of parents. The scores range from 23.33 to 100, with higher scores meaning higher anxiety. Anesthesia was induced with oxygen/N₂O, and sevoflurane was administered via a transparent face mask.

Data analysis

Data were entered and analyzed using IBM SPSS Statistics for Windows, Version 19 (Released 2010; IBM Corp., Armonk, New York). To summarize the characteristics of data, mean (SD)/median (IQR) were computed for numerical variables (normal/non-normal) such as age and mYPAS score, while frequencies (percentages) were reported for categorical variables such as gender and ASA (American Society of Anesthesiologists) status. To examine the shape of the continuous variable's distribution, the Shapiro-Wilk test was used (normality assumption). To determine any statistically significant differences between the two groups, an independent t-test/Mann-Whitney U test was used for anxiety scores, while a chi-square or exact Fisher's test was used for gender, ASA status, and age groups, respectively. The Mann-Whitney U test was used to compare the anxiety scores of the two groups. A p-value less than 5% was considered statistically significant.

## Results

A total of 106 children were included in the study. Table 1 shows the demographic data of the two groups, indicating that the majority of the patients (61.3%) were aged between two and four years, while 82% were males.

**Table 1:** Patient demographics ASA: American Society of Anesthesiologists

Variables	Total (n=106)	Group A (n=53)	Group B (n=53)	p-value
Age groups (years)				
2–4, n (%)	65 (61.3%)	30 (56.6%)	35 (66%)	0.057
5–12, n (%)	39 (36.8%)	22 (41.5%)	17 (32.1%)	0.332
13–18, n (%)	2 (1.9%)	1 (1.9%)	1 (1.9%)	1.000
Gender				
Male, n (%)	87 (82.1%)	45 (84.9%)	42 (79.2%)	0.447
Female, n (%)	19 (17.9%)	8 (15.1%)	11 (20.8%)	
ASA status				
I, n (%)	84 (79.2%)	39 (73.5%)	45 (84.9%)	0.151
II, n (%)	22 (20.8%)	14 (26.4%)	8 (15%)	

Table 2 shows the mYPAS scores for both groups. The minimum anxiety scores in the preoperative area were 23.33 and a maximum of 78.33, while at induction, the minimum scores were 23.33 and a maximum of 70.00. The maximum scores in Group A were 70.0 (median 46.67, IQR 26.6), while in Group B, they were 78.33 (median 28.33, IQR 23.33) preoperatively. In contrast, at induction, Group A was 68.33 (median 46.67, IQR 27.50) and 55.0 (median 23.33, IQR 10.0) in Group B.

**Table 2:** Modified Yale Preoperative Anxiety Scale (mYPAS) scores IQR: interquartile range; OR: operating room

Modified Yale Preoperative Anxiety Scale Score	Groups	Minimum	Maximum	Median (IQR)
At preop	Overall	23.33	78.33	33.33 (28.33)
	A	23.33	70.0	46.67 (26.67)
	B	23.33	78.33	28.33 (23.33)
At OR	Overall	23.33	70.0	31.67 (25.42)
	A	23.33	68.33	46.67 (27.50)
	B	23.33	55.0	23.33 (10.0)

Table 3 shows the comparison of Groups A and B using the Mann-Whitney U test. The Shapiro-Wilk test was applied to examine the normality of average scores at preop and OR, and the distributions were found to be significantly different from normal with a p-value < 0.001. Therefore, the Mann-Whitney U test was used to compare Groups A and B, and it showed that Group B experienced less anxiety (43.84 vs. 62.34) compared to Group A, both in the preop area and inside the OR (939.74 vs. 63.73).

**Table 3:** Comparison of Groups A and B using the Mann-Whitney U test *Mann-Whitney U test: p-value < 0.05 was considered statistically significant.

Modified Yale Preoperative Anxiety Scale	Mean Ranks			p-value*
	Group A (n=53)	Group B (n=53)	95% Confidence interval (L, U)	
Average score at preop	62.34	43.84	(582.31, 1202.69)	0.001
Average score at OR	63.73	39.74	(378.31, 998.69)	< 0.001

The subgroup analysis was performed to check the difference between the age groups. Table 4 shows the anxiety scores of children between the ages of two and four, revealing the statistical significance between the two groups in the preop area and just before induction in the OR (p = 0.0001). There was not much difference in the anxiety scores of children aged between five and 12 years preoperatively, but they were statistically significant just before induction (p = 0.03), as shown in Table 5.

**Table 4:** Modified Yale Preoperative Anxiety Scale (mYPAS) scores (ages two to four years)

Modified Yale Preoperative Anxiety Scale	Groups	Minimum	Maximum	Median (IQR)	p-value
At preop	A	23.33	70.0	50 (23.33)	0.0001
	B	23.33	65.0	48.33 (28.34)	
At OR	A	23.33	78.33	25.83 (23.34)	0.0001
	B	23.03	70.0	23.33 (10.0)	

**Table 5:** Modified Yale Preoperative Anxiety Scale (mYPAS) scores (ages five to 12 years)

Modified Yale Preoperative Anxiety Scale	Groups	Minimum	Maximum	Median (IQR)	p-value
At preop	A	23.33	61.67	43.33 (26.67)	0.1025
	B	23.2	68.33	45 (31.67)	
At OR	A	23.33	65.0	28.33 (10.0)	0.0386
	B	23.33	46.67	28.33 (12.05)	

**Figure 1:**
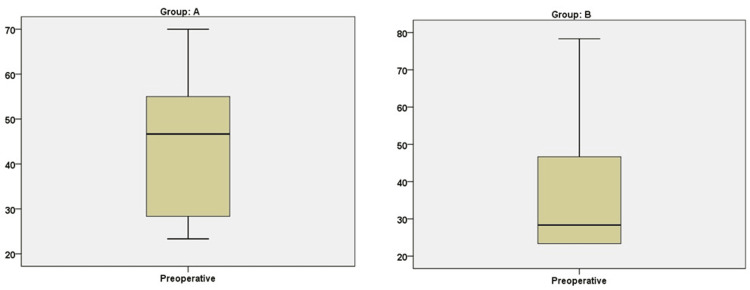
Group comparison of the Modified Yale Preoperative Anxiety Scale (mYPAS) score at the preoperative stage

**Figure 2:**
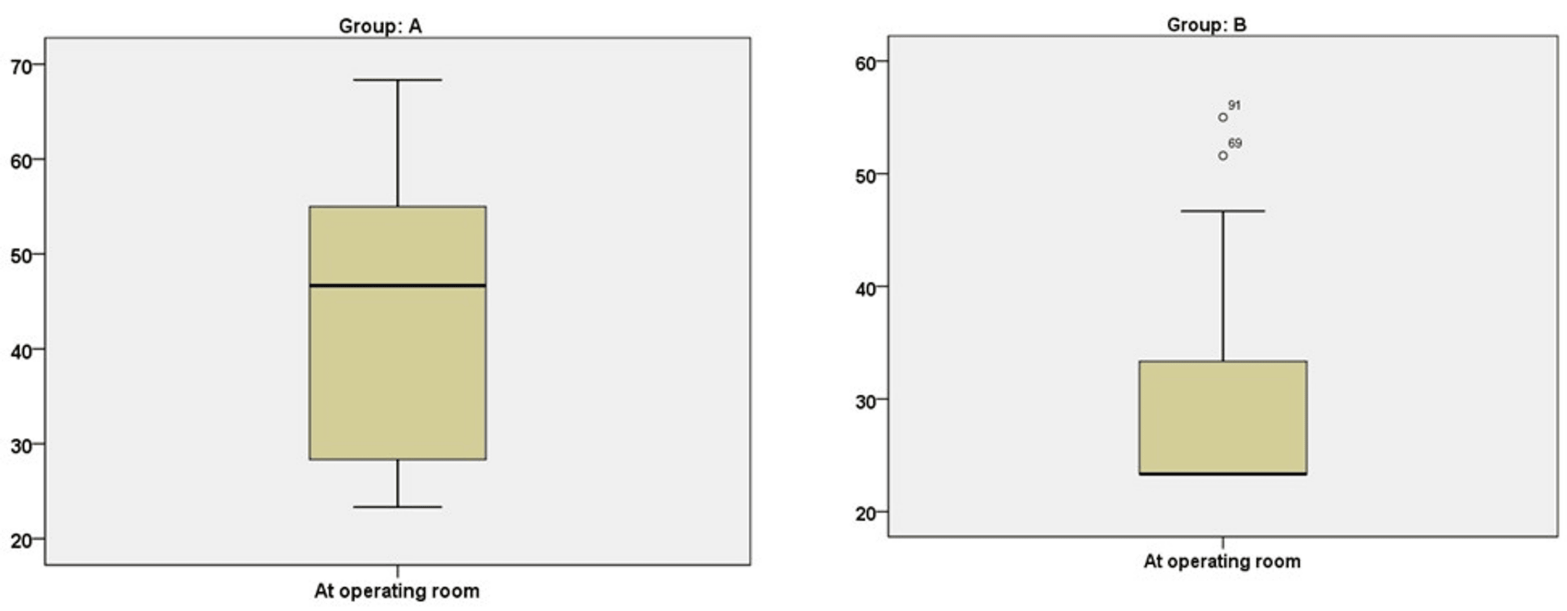
Group comparison of the Modified Yale Preoperative Anxiety Scale (mYPAS) score in the operating room

Figures 1, 2 show the group comparison of the mYPAS score at preoperative and intraoperative times, respectively.

## Discussion

The study effectively evaluated the effect of nonpharmacological interventions, i.e., the use of digital devices, in combating preoperative anxiety in children undergoing surgeries, as shown by the results.

Pharmacological methods, including premedication, have been used to address preoperative anxiety. Midazolam, as a premedication, is a reliable strategy for reducing preoperative anxiety [8]. However, strict monitoring must be maintained, as it may result in high levels of impulsivity and delayed anesthesia emergence [5].

Nonpharmacological interventions are minimally invasive and cost-effective, with fewer adverse effects, and therefore can have widespread implementation [9]. The examination of the literature on nonpharmacologic strategies for reducing anxiety prior to surgery revealed a variety of approaches that have been tested for this purpose and produced encouraging outcomes [10]. However, there is a clear paucity in the current literature on reducing preoperative anxiety in pediatric populations. In pediatric patients, especially younger ones for whom parental separation is likely to be a source of stress and anxiety, environmental factors like the availability of appropriate games and toys, augmented and virtual reality, and parental presence are important factors in reducing anxiety [11]. To implement these techniques in clinical settings, there are certain reservations regarding their accessibility, expense, and need for highly qualified staff [3,11].

There are a few studies from other countries published to overcome children's anxiety during the perioperative period [12,13]. However, there are numerous differences between Pakistan and other countries concerning the growth environment, educational concepts, and children's relationships with their parents. Therefore, the research experience of other countries may not be relevant to this region. There is also a paucity of adequately powered randomized controlled trials to confirm the usefulness of nonpharmacological interventions in Pakistani children. Therefore, these findings can be added to the literature regarding nonpharmacological methods for treating and preventing preoperative anxiety in young patients from low- and middle-income countries.

Based on the mYPAS scores, it has been seen that the anxiety of children increases progressively from the holding area to the transport to the mask introduction [9]. Children who watch or play video games on streaming services are completely oblivious to their surroundings. Video distraction is a better source of diversion when inhaling anesthesia. Similar results were also observed in Lee et al.'s study, which found that preschoolers who were accompanied by their parents throughout the perioperative phase experienced less anxiety due to cartoon distraction than the control group [14]. In another study, Mifflin et al. found that children in the control group experienced a higher rise in anxiety from the holding area to induction than did those in the video distraction group [15]. In our study, one of the parents accompanied the child to the OR in both groups; however, parental presence alone was not associated with decreased anxiety, as shown in the results.

Another important discovery we made was that none of our patients, including those who lived in rural parts of the nation, needed to be taught how to use tablets or other digital devices. The younger generation is therefore technologically adept, and in high-workload situations when premedication might not be an option owing to a lack of monitoring time, video distraction can be a helpful solution. However, further research is needed to better understand its effectiveness across different age groups, surgical procedures, and settings [16,17].

Furthermore, it is critical to remember that creating a friendly atmosphere and communicating with patients and their parents about the treatment are tried-and-true strategies for reducing patients' levels of tension and anxiety [18]. Video distraction can improve all these techniques and should be used along with these to reduce the preoperative anxiety of the children [18].

The strengths of this study include the randomized controlled design, utility, and the use of an existing and validated behavioral-based scale to reduce observer bias. Moreover, the study's broad inclusion criteria and diverse case selection may enhance its relevance and provide insights that could be applicable to a wider range of pediatric patients.

This study had certain limitations as well. First off, we are unable to comment on whether the video distraction intervention was effective in reducing postoperative anxiety and maladaptive behaviors because the children were not followed up in the postoperative period. Secondly, observer bias cannot be completely ruled out because the data collector was unable to be blinded for evident reasons. Additionally, the study focused on behavioral anxiety and did not take into account the sedation levels, cardiovascular parameters, and baseline anxiety of the patients. Finally, because this study was conducted at Pakistan's largest private hospital, it is not representative of the country's whole population.

We recommend that the focus of future research should involve a longer postoperative follow-up and the addition of more areas where children's procedures are performed, such as radiological procedures and treatments outside the ORs.

We can conclude that video distraction is a useful nonpharmacological treatment for reducing children's anxiety during the preoperative phase. Moreover, using this technique to reduce anxiety in children undergoing elective surgery could enhance their hospital experience overall by increasing their cooperation with the medical staff before surgery. Furthermore, the technology is quite simple and easy to apply in clinical practice, requiring no additional labor or higher costs for healthcare services. This modality ensures emotional and mental well-being through anxiety reduction and may have a positive effect on expediting recovery. It may also be beneficial for healthcare personnel who are responsible for providing care for children and their families during the challenging stages of the perioperative process, which includes the OR.

## Conclusions

Nonpharmacological intervention with video distraction in comparison to pharmacological intervention reduces preoperative anxiety, as indicated by the mYPAS scores. Therefore, routine video distraction is a low-cost, simple-to-implement, and effective nonpharmacological method to reduce anxiety in the preoperative period and right before the induction of anesthesia in settings with a high patient turnover rate.
